# Application of Artificial Intelligence Tools for Social and Psychological Enhancement of Students with Autism Spectrum Disorder: A Systematic Review

**DOI:** 10.3390/brainsci16010056

**Published:** 2025-12-30

**Authors:** Angeliki Tsapanou, Anastasia Bouka, Angeliki Papadopoulou, Christina Vamvatsikou, Dionisia Mikrouli, Eirini Theofila, Kassandra Dionysopoulou, Konstantina Kortseli, Panagiota Lytaki, Theoni Myrto Spyridonidi, Panagiotis Plotas

**Affiliations:** 1 Department of Speech Therapy, University of Patras, 26504 Patras, Greece; tsapanou@upatras.gr; 2 Postgraduate Program in Health Education, University of Patras, 26504 Patras, Greece; andbouka@gmail.com (A.B.); papadopoulouangelik@gmail.com (A.P.); c.vamvatsikou@gmail.com (C.V.); dion.mikrouli@gmail.com (D.M.); up1118781@upatras.gr (E.T.); kassandrad19@gmail.com (K.D.); up1118629@upatras.gr (K.K.); p.lytaki@gmail.com (P.L.); theonimyrtospyridonidi@gmail.com (T.M.S.); 3 Primary Healthcare Laboratory, School of Health Rehabilitation Sciences, University of Patras, 26504 Patras, Greece

**Keywords:** children, autism spectrum disorder, social skills, AI tools, interaction, generative artificial intelligent

## Abstract

Background: Children with autism spectrum disorder (ASD) commonly experience persistent difficulties in social communication, emotional regulation, and social engagement. In recent years, artificial intelligence (AI)-based technologies, particularly socially assistive robots and intelligent sensing systems, have been explored as complementary tools to support psychosocial interventions in this population. Objective: This systematic review aimed to critically evaluate recent evidence on the effectiveness of AI-based interventions in improving social, emotional, and cognitive functioning in children with ASD. Methods: A systematic literature search was conducted in PubMed following PRISMA guidelines, targeting English-language studies published between 2020 and 2025. Eligible studies involved children with ASD and implemented AI-driven tools within therapeutic or educational settings. Eight studies met inclusion criteria and were analyzed using the PICO framework. Results: The reviewed interventions included humanoid and non-humanoid robots, gaze-tracking systems, and theory of mind-oriented applications. Across studies, AI-based interventions were associated with improvements in joint attention, social communication and reciprocity, emotion recognition and regulation, theory of mind, and task engagement. Outcomes were assessed using standardized behavioral measures, observational coding, parent or therapist reports, and physiological or sensor-based indices. However, the studies were characterized by small and heterogeneous samples, short intervention durations, and variability in outcome measures. Conclusions: Current evidence suggests that AI-based systems may serve as valuable adjuncts to conventional interventions for children with ASD, particularly for supporting structured social and emotional skill development. Nonetheless, methodological limitations and limited long-term data underscore the need for larger, multi-site trials with standardized protocols to better establish efficacy, generalizability, and ethical integration into clinical practice.

## 1. Introduction

Autism spectrum disorder (ASD), hereafter referred to as autism, originates from the Greek word “autos”, which is synonymous with “self-isolated” or “automatic movement.” [1]. Children with autism spectrum disorder (ASD) experience persistent difficulties in social communication, emotional regulation, and social participation, which often limit the effectiveness of traditional intervention approaches that rely heavily on complex human social cues. Although evidence-based behavioral and educational therapies remain the cornerstone of treatment, these approaches can be resource-intensive, difficult to individualize, and challenging for children who experience heightened social anxiety or sensory overload. In this context, artificial intelligence (AI)-based technologies, particularly socially assistive robots, have emerged as promising complementary tools due to their predictable behavior, consistency, and capacity for personalization. While an increasing number of studies report the beneficial effects of AI-assisted interventions in ASD, findings remain fragmented across disciplines, robot platforms, and outcome domains. To date, there is a lack of integrative synthesis focusing on how and in which domains these technologies contribute to psychosocial improvement. The present systematic review addresses this gap by critically evaluating recent AI-based interventions targeting social, emotional, and cognitive functioning in children with ASD. The altered expression of specific genes causes ASD [2]. The Centers for Disease Control and Prevention (CDC) defines ASD as a developmental disability that causes significant challenges in social interaction, communication, and behavior [3]. Leo Kanner, an Austrian-American psychiatrist and social activist, was the first to attempt to describe ASD. In 1943, he claimed that autism was a disorder characterized by problems relating to others and a high sensitivity to changes in the environment [4]. Autism spectrum disorder started as a rare disease, but it has become increasingly prevalent. Since 2000, the Autism and Developmental Disabilities Monitoring (ADDM) Network has reported biennial estimates of the prevalence of ASD among eight-year-old children. The prevalence of ASD has increased from one in a hundred-and-fifty in 2000 [5] to one in thirty-six in 2020 [6]. This increase may be due to the evolving criteria prior to the publication of the Diagnostic and Statistical Manual of Mental Disorders, 5th Edition (DSM-5) [7], as well as increased awareness by society and the mandatory availability of treatments [8].

It is imperative to acknowledge that the characteristics of ASD are subject to variation and may not always manifest in the same manner or at the same age in children. However, there are common features frequently found in children with the disorder. These include impairments in social and communication skills, as well as repetitive patterns of behavior, interests, or activities [9]. Children with this disorder tend to isolate themselves from social interactions [10]. In addition, researchers have observed specific behaviors such as hyper-vigilance, aggression [11], as well as developmental delay and cognitive impairment [12]. Furthermore, the issue of inadequate eye contact should be considered as well as limited or non-functional play skills and difficulty expressing and understanding emotions [13]. Enhancing sociality in autistic children is essential because through social participation, they will actively take part in social activities and acquire social roles. They will integrate smoothly into the community.

Research has indicated that children diagnosed with ASD have shown improvement in social skills, including communication, imitation, and attention when engaging with robots as part of their treatment [14]. AI technologies have been functioning as tools in the treatment of this population. Social-assistive robots (SARs) have recently been included in treatments for ASD as they capture the interest of children. Their simple, repetitive movements and appearance make it easier for children to show confidence [15]. The physical appearance of SARs facilitates interaction with this population. Humanoid robots successfully mimic the appearance of the human body. They are characterized by eye contact, verbal communication (enriched with critical speech, e.g., praise), and movement with natural gestures. These features act as stimuli that aid the treatment process (e.g., speech enhancement) and help reduce anxiety associated with learning [16]. According to the literature, robotic intervention augments joint attention [17], motivation [18], and social skills [19]. Research studies [20] have shown that the robotic intervention group demonstrated the greatest improvement in performance. These findings claim that robotic intervention is the most effective method for improving social communication and the behavior of children with ASD.

The archive has been further enriched by the inclusion of robots in animal form, thus offering a range of topics for exploration. Robots that resemble animals are becoming increasingly common in research, as animals are utilized within a variety of educational and therapeutic settings [21]. Research by [22] has demonstrated the usefulness of therapies carried out with animals as a popular intervention for children with developmental difficulties, such as autism [22]. It is evident that advanced robot animals have been developed to the extent that they are interactive enough and can perform similar actions as live animals, e.g., autonomous behavior, obeying commands, showing emotions, and following children around the room [23].

The research framework is supported and reinforced by several theoretical foundations. The study by [24], for instance, has been grounded in the theoretical underpinnings of the Theory of Mind (ToM), which is a pivotal mechanism of social cognition. The research protocol employed in this study involved the utilization of a visual attention tracking system, a methodological approach that has been instrumental in elucidating the intricacies of social cognitive processes.

In the context of emotional support for children diagnosed with autism, it has been widely accepted that these children demonstrate a more positive emotional response when engaging with robots [9]. Moreover, the efficacy of empathetic actions is enhanced when performed with robots as opposed to humans [25]. A growing body of empirical studies has reported positive effects of AI-assisted interventions on social, emotional, and attentional outcomes in children with ASD. However, this literature remains fragmented across disciplines, intervention designs, robotic platforms, and outcome measures.

Therefore, the aim of the present systematic review is to critically evaluate recent studies examining AI-based interventions for children with ASD, with a particular focus on their effects on social communication, emotional functioning, attention, and social cognition. By synthesizing evidence from the last five years, this review seeks to clarify the domains in which AI tools appear most beneficial, highlight methodological limitations of existing studies, and identify key directions for future research and clinical application.

## 2. Methodology

The literature search for this study was based on a systematic review of research study articles related to our research hypothesis according to the PRISMA (Preferred Reporting Items for Systematic Reviews and Meta Analysis) statement, which provides a comprehensive guideline for conducting systematic reviews (see Supplementary Materials). For the present study, a search strategy was developed to identify studies investigating the effectiveness of artificial intelligence tools in enhancing the social and psychological well-being of students with ASD.

The search was conducted in the PubMed (http://www.ncbi.nlm.nih.gov/pubmed) online database, using MESH terms, i.e., “children, “autism spectrum disorder”, “improvement”, “social skills”, “communication”, “interaction”, “robotics”, “artificial intelligent”, “Ai tools”, “generative artificial intelligent”, accessed on 5 May 2025. Boolean language operators (AND, OR) were also used for an efficient search in combination with MESH terms, i.e., ((children) AND (autism spectrum disorder)) AND (((improvement) AND (social skills)) OR (communication)) OR (interaction))) AND ((((robotics) OR (artificial intelligence)) OR (Ai tools)) OR (generative artificial intelligence)).

The search was limited to articles published in English, while the search dates were set to the last five years, i.e., from 2020 onwards, to include the most recent literature.

## 3. Exclusion Stages and Criteria

The inclusion criteria were studies conducted around the world, in English, on children. The initial search yielded 65 articles on PubMed. Every title and abstract of each study was checked and those that did not meet the inclusion criteria were excluded. After reviewing the remaining studies, eight were selected for the final sample.

**Figure 1:**
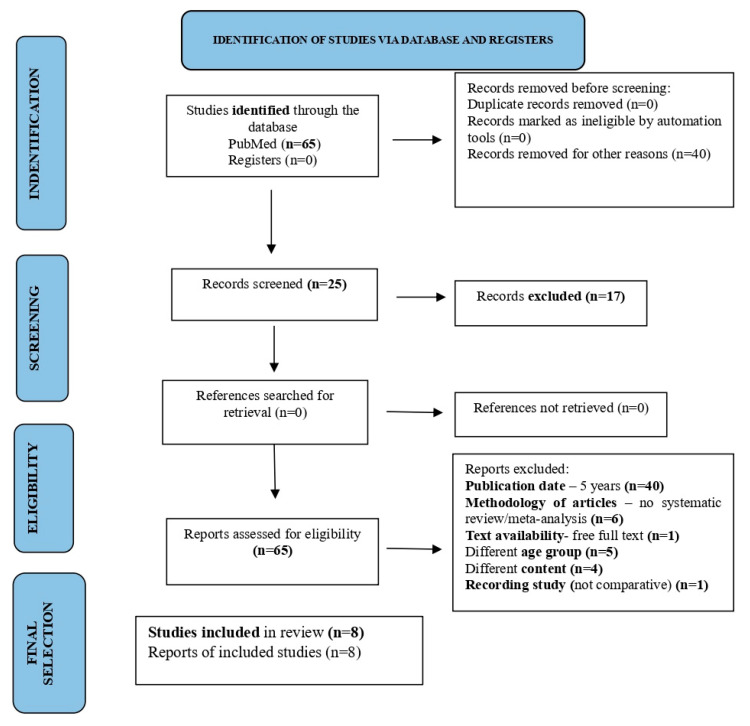
PRISMA flowchart for the selection of studies on the application of AI tools for the social and psychological support of students with ASD.

The following PRISMA diagram below illustrates the process of selecting articles for this review (see Figure 1).

## 4. Results

Characteristics of studies included the following:

The characteristics of the studies described in this section are based on eight studies. Most of the studies were RCTs (*n* = 62.5%) and the rest were non-randomized (*n* = 37.5%). On average, the sample size of the students with ASD in the studies was 36 (M = 35.875).

In the studies that specified the gender of the participants (75%), the participants were predominantly male, with percentages varying from 61.5% to 90%. The ethnicity of the participants was explicitly stated in three studies (37.5%), with Dutch [23], Italian [24], Japanese, and French [25]. A total of 18 participants were included. Τhe ages of the participants ranged from 5 years to 16 years.

The process of diagnosing autism was specified in most of the studies, with two of the studies providing further information about the tools used in the diagnosis (25%), and five reassessing ASD prior to intervention (62.5%). The intelligence quotient (IQ) score of the participants was only provided in three studies (37.5%) and stated in the inclusion criteria of two others (25%). The studies were published between 2020 and 2025, predominantly in Europe (62.5%), i.e., France (and Japan), Greece, Italy, the Netherlands, and Portugal, followed by two in Hong Kong [20]. So and colleagues [26] published the one in Colombia [27] (see Table 1).

## 5. Settings

The sessions of each intervention were conducted in eight different settings. Three were conducted in schools, followed by home, a host university, a rehabilitation center, a pediatrics clinic, a learning disabilities center, a community-based educational setting, and the physiotherapy room of a clinic. The mean duration of the sessions involving robot intervention was 25 min, with the sessions reaching an average of nine for the duration of each study. The sessions took place once or twice a week, according to the studies disclosing this information (75%).

## 6. Interventions

All the studies used a type of robot to implement their intervention. Most of the studies (75%) used humanoid robots, resembling a human body, with facial characteristics present as well. The humanoid robots used in the sessions were the NAO, iCUB, CASTOR, and HUMANE robots. The use of an animaloid (animal resembling) robot was recorded in one of the studies [23]. The robot used, which resembled a dog, was the WowWee CHiP robot dog. In this study, the results of robot dog-assisted therapy were compared to results from dog-assisted therapy for children with ASD and Down syndrome. Lastly, a study used a toy robot [24] and another used a plant robot [25]. The toy robot, called Cozmo, is a commercial toy robot. The plant robot, called “Pekkopa”, is designed to react to sounds of speech and provide nodding responses.

The interventions were built around the use of the previously mentioned robots as social facilitators for children with ASD. Their primary focus was targeting the behavior and use of social and emotional skills, such as, attention, communication, social participation, and cognition, regulating and identifying emotions and emotional empathy.

Humanoid robots, particularly NAO, were employed on the basis that human-like appearance, gestures, gaze direction, and speech would add a sense of familiarity to the participants. This facilitated its use in engaging children in performance-based social communication tasks [20]. Holeva et al. [28] employed NAO to facilitate Theory of mind tasks and emotion recognition exercises, while NAO and the non-intrusive gaze-tracking software, Gaze360, were used to assess joint attention during therapy [29]. The CASTOR robot supported the sessions by providing verbal prompts and visual stimuli to engage the children’s attention, memory, physical, verbal imitation, and emotional recognition [24] and was compared to a humanoid robot, iCUB, with a non-humanoid robot, Cozmo, in an intervention targeting the development of the theory of mind. A robot drama paradigm was implemented by So et al. (2020) [26], in which two HUMANE robots performed scripted scenes in an effort to improve the joint attention of the participants.

On the contrary, the use of an animaloid robot occurred in [23], where the introduction of a robotic dog in dog-assisted therapy was compared to dog-assisted therapy with a live dog. In both groups, children practiced emotion recognition and self-confidence exercises, while targeting the improvement of their social skills. Lastly, ref. [25] 18 participants compared interpersonal synchronization and emotional empathy, on a cross-cultural scale, employing the minimalistic nodding robot, “Pekkopa”, which resembled a plant. Interaction with the robot was compared to human interaction and was analyzed based on autonomic reactions, such as heart rate and heart rate variability.

## 7. Outcomes

Across studies, intervention effects were evaluated using standardized behavioral and neuropsychological assessments, structured observational coding, parent- and therapist-report questionnaires, and objective physiological or sensor-based measures (e.g., gaze-tracking and heart rate variability), with improvements defined as statistically significant gains or maintained performance in the targeted domains.

All the studies demonstrated that the robot-assisted interventions yielded positive, even though varied, outcomes in children with ASD. Robot-assisted intervention led to significant improvements in response to joint attention (RJA) and initiation of joint attention (IJA) as reported in So et al. (2020) [26], while Silva [29] noted an improvement of the overall joint attention, but a gradual decline in the attention paid to the NAO robot. For Holeva et al. (2022) [28], an overall improvement of psychosocial skills was present in both robot-assisted and human-only groups, with enhanced social interactions occurring in the robot-assisted group. In a similar pattern, robotic intervention led to greater improvements in social communication compared to human-led and control groups for [20]. The humanoid robot iCUB significantly improved theory of mind, as opposed to the Cozmo robot, which did not yield such results [24]. Improvement or maintenance of social skills was a result of the intervention with the CASTOR robot [27]. A greater improvement of emotional attunement and emotion regulation was correlated to dog-assisted therapy, in comparison to robot dog-assisted therapy, with effects on other social skills not differing significantly [23]. For [25], autonomic synchronization and emotions were improved for all participants when interacting with the robot, rather than the human partner. Empathetic reactions and heart rate differed amongst the participants from differed countries. For the measurement of targeted skills, three studies made use of standardized assessment tools: [20,24]; Holeva et al. (2022) [28].

In addition, ref. [20] made use of parent-reported questionnaires, as did [23]. Therapists’ recordings during the sessions were utilized by Holeva et al. (2022) [28], So et al. (2020) [26] relied on video recordings of the sessions, while Gaitan–Padilla, Cifuentes and Munera (2022) [27] made use of the therapists’ evaluations. Measurements using gaze-tracking technology were extracted to evaluate joint attention [24]. Lastly, ref. [25] 18 used heart rate and heart rate variability measurements, as well as emotional reports after each session (see Table 2).

## 8. Discussion

**Figure 2:**
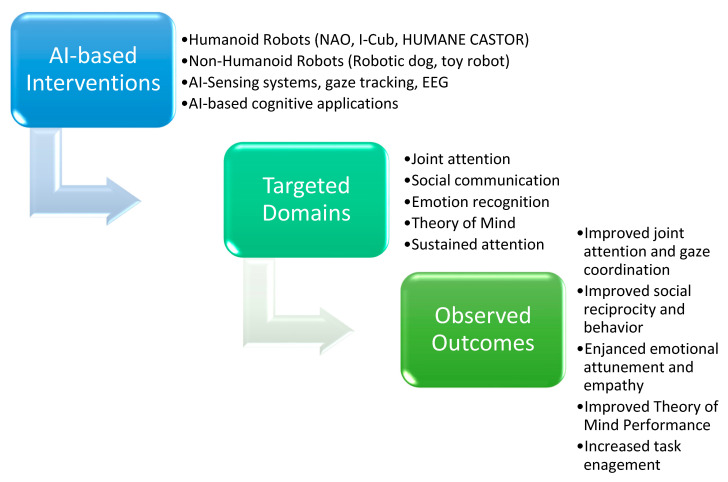
Intervention: outcome map of AI-based tools in ASD.

This systematic review highlights the growing academic interest in applying artificial intelligence (AI)-based interventions in socially assistive robotics (SAR) to support children with autism spectrum disorder (ASD) and related neurodevelopmental conditions. The five included studies, which were conducted in various geographical locations (the Netherlands, Portugal, Italy, Hong Kong, and Greece), primarily employed rigorous methodological designs, with the most notable being randomized controlled trials (RCTs), and collectively provided valuable evidence regarding the clinical efficacy and practical utility of these technologies (see Figure 2).

A notable finding across the studies was the prevalence of humanoid robotic platforms, particularly the NAO robot and the iCub, with only one study using a non-humanoid robotic dog. These robotic systems were embedded within structured, therapeutic, or educational interventions aimed at improving core deficits in social communication, emotional regulation, and theory of mind.

One particularly notable study in the Netherlands [23], featured an innovative application of a robotic dog with a diverse group of 65 children with autism or Down syndrome. The intervention led to observable improvements in emotional alignment and regulation, with an emphasis on individualized interaction patterns. Despite its strengths, including active control condition and a sizable sample, the reliance on self-report measures and the heterogeneity of the clinical population limited the generalizability of the findings.

By contrast, the study by Ghiglino [24], carried out in Italy, offered a more targeted exploration of socio-cognitive enhancement. This randomized controlled trial (RCT) involved 45 children and compared a humanoid robot (iCub) with a non-humanoid robotic alternative. Significantly greater improvement in theory of mind, emotional comprehension, and prosocial behaviors were observed in the iCub condition. The use of validated instruments, such as the NEPSY-II, strengthened the reliability of the results. However, the relatively high IQ of the participants may have introduced a selection bias which could affect the external validity of the findings.

The Greek study involved the use of the NAO robot in a psychoeducational social reinforcement program with 51 children aged 6–12 with ASD [28]. While both the experimental and control groups showed improvement, the robotic-enhanced intervention was particularly effective in enhancing emotion recognition skills. The study was methodologically robust. However, the long-term impact remains unclear due to the absence of follow-up assessments, largely due to constraints related to the pandemic.

The study by Silva et al. (2024) [29], conducted in Portugal and Italy, introduced a technically sophisticated AI framework to monitor social attention via non-invasive EEG sensors. Despite its small sample size (*n* = 5), the system achieved a classification accuracy of 72.5%, suggesting significant potential for the real-time personalization of SAR-based interventions. However, the exploratory nature of the study and technical limitations in EEG signal acquisition necessitate cautious interpretation. Chung et al. (2025) [20] worked with a larger cohort of 60 children in Hong Kong and implemented the NAO robot to foster improvements in social reciprocity and communication. Compared to the control group, the intervention group exhibited statistically significant improvements in initiating social interactions and reducing non-functional behaviors. This finding further supports the idea that humanoid SAR systems can enhance foundational social competencies across cultures.

Overall, the studies support the idea that AI interventions can improve the social and communication skills of children with ASD. Emphasis is placed on the anthropomorphic nature of robots, personalized interventions, and integrated advance monitoring systems. In this review, “improvement” refers to specific gains in the targeted psychosocial domain, rather than a global change in functioning. Across studies, AI-based interventions were associated with improvements in joint attention, social communication, reciprocity, emotion recognition and regulation, theory of mind, attention/engagement, and physiological indices. Further research should focus on multicenter studies with sufficient statistical power, as well as the use of mixed methods and the integration of biomarkers to understand the neurobiological mechanisms associated with intervention success. At the same time, acceptance by families and the ethics of using robots with children should be systematically investigated.

The reported improvements were achieved through structured, goal-oriented AI-based interventions, primarily involving socially assistive robots (humanoid, animal-like, or minimalistic) used as interactive mediators during therapy or educational sessions. These interventions typically included guided social communication tasks, joint-attention exercises, emotion recognition and regulation activities, imitation and turn-taking games, and Theory of Mind scenarios, often supported by real-time feedback, gaze-tracking, or physiological monitoring. The predictable, responsive, and engaging nature of these AI systems was central to facilitating the observed improvements in social, emotional, and attentional domains.

The anthropomorphic features of humanoid robots probably make it easier to establish natural social interactions, which may partly explain the superior efficacy. However, the innovative use of non-humanoid robots, such as robotic dogs, suggests a distinct mechanism of affective engagement that is particularly valuable for populations with diverse neurodevelopmental profiles.

Despite the encouraging findings, the included studies exhibited notable limitations, including small and heterogeneous samples, short intervention durations, lack of long-term follow-up, variability in outcome measures, limited use of standardized assessments, and potential novelty or expectancy effects associated with robot-assisted interventions. The relatively small number of included studies likely reflects the strict inclusion criteria and the early stage of research on AI-based psychosocial interventions for children with ASD, which should be considered when interpreting the findings. The technological complexity and the cost of SAR systems also pose practical constraints to scalability. A limitation of this review is also the reliance on PubMed as the sole database, which may have excluded relevant interdisciplinary studies from engineering, computer science, and educational databases; therefore, future reviews should adopt a multi-database search strategy to ensure more comprehensive coverage.

Although the results appear highly promising, this review also identifies several strengths and limitations. A key strength lies in the inclusion of studies conducted across diverse cultural and geographical contexts, as well as the emphasis on methodologically rigorous designs set on randomized controlled trials. This diversity enhances the reliability and ecological validity of the findings. However, the limited number of available studies, as well as small and heterogeneous samples in the lack of long-term follow-up data, restricts the generalizability of the results. Addressing these limitations in future research will be essential for developing more reliable and scalable AI-based interventions in the field of socially assistive robotics.

Beyond the overall positive outcomes, the findings suggest that intervention effects may be partially explained by the specific characteristics of AI-based systems. Humanoid robots, which provide gaze direction, verbal feedback, and human-like gestures, were more consistently associated with improvements in higher-order social cognition, such as theory of mind and emotion understanding, whereas non-humanoid robots appeared to primarily support affective engagement and emotional regulation. The predictable, structured, and non-judgmental nature of AI-mediated interactions may reduce social anxiety and cognitive load in children with ASD, thereby facilitating engagement and learning. However, the considerable heterogeneity in robot platforms, intervention intensity, outcome measures, and sample characteristics limits direct comparability across studies and underscores the need for more standardized, mechanism-driven research.

## 9. Conclusions

This review highlights the growing use of artificial intelligence (AI) in helping children with Autism Spectrum Disorder (ASD) develop social, emotional, and thinking skills. The studies reviewed used different kinds of AI tools, such as social robots, humanoid assistants, gaze-tracking systems, and programs designed to train Theory of Mind (ToM) abilities. Overall, robot-based activities showed clear benefits in joint attention, imitation, and emotional connection, while other AI systems also gave positive results. Tools that tracked children’s eye gaze helped therapists understand how engaged the children were, matching what they observed during sessions. Likewise, AI programs that supported ToM skills improved social understanding and reasoning in a friendly and less stressful way. These technologies seem most effective when used in structured and personalized sessions that fit each child’s needs. Their predictable and responsive design helps children, especially those with more severe autism, take part in social activities that might otherwise feel difficult. However, many studies had small groups of participants, short durations, and different ways of measuring progress, which make it harder to compare results. Despite these challenges, the overall findings suggest that AI-based tools can work well alongside traditional therapies. Future studies should explore their long-term impact, how children can use these skills in daily life, and how to apply such systems safely, fairly, and in ways that support inclusion.

## Figures and Tables

**Table 1:** Integrated summary of study characteristics, interventions, and outcomes across the included studies.

**Year**	**Author**	**Title**	**Country**	**Study Design**	**Sample Size**	**Age Group**	**Population Characteristics**
2020	So et al. [26]	Effectiveness of a Robot Drama Paradigm on Joint Attention in Children with ASD	Hong Kong	Randomized controlled trial	30	6–12 years	Children diagnosed with ASD
2018	Giannopulu et al. [25]	Emotional Empathy as a Mechanism of Synchronisation in Child-Robot Interaction	France and Japan	Experimental cross-cultural study	40	6–15 years	Children with ASD
2022	Holeva et al. [28]	Robot-Assisted Psychoeducational Intervention for Children with ASD	Greece	Randomized controlled trial	51	6–12 years	Children with ASD, IQ within normal range
2022	Gaitán-Padilla et al. [27]	Socially-Assistive Robotics for Improving Social Participation in ASD	Colombia	Non-randomized intervention study	20	5–10 years	Children with ASD
2023	Ghiglino et al. [24]	Artificial Scaffolding of Social Cognition Using Humanoid Robots	Italy	Randomized controlled trial	45	7–14 years	Children with ASD, moderate–high IQ
2024	Chung, Sin and Chow [20]	Robotic Intervention to Improve Social Communication in ASD	Hong Kong	Randomized controlled trial	60	6–10 years	Children with ASD
2024	Silva et al. [29]	AI-Based Gaze and EEG Monitoring During Robot-Assisted Therapy	Portugal and Italy	Pilot exploratory study	5	8–16 years	Children with ASD
2025	Van der Steen et al. [23]	Dog-Assisted vs. Robot Dog-Assisted Therapy in Neurodevelopmental Disorders	Netherlands	Randomized controlled trial	65	6–14 years	Children with ASD and Down syndrome
**AI Intervention Type**		**Outcome Domain**	**Key Findings**		**Strengths**		**Limitations**
Humanoid robots (e.g., NAO, iCub, HUMANE, CASTOR)		Joint attention, social communication	Improvements in joint attention, social reciprocity, and emotion recognition		High engagement; structured and predictable interaction		Small samples; limited long-term follow-up
Humanoid robots (iCub)		Theory of mind	Improved theory of mind and prosocial behaviors		Use of standardized assessments		Participants are often high-functioning; limited generalizability
Non-humanoid robots (robotic dog, toy/plant robot)		Emotional regulation, empathy	Enhanced emotional attunement and affective engagement		Reduced social complexity; lower anxiety		Mixed effectiveness compared to live agents
AI sensing systems (gaze-tracking, EEG, HR/HRV)		Attention, engagement	Increased task engagement and attention during sessions		Objective, real-time measurements		Technical complexity; very small samples
AI-based cognitive applications (ToM tasks)		Social cognition	Improved social understanding in structured settings		Targeted cognitive training		Limited evidence for transfer to daily life

**Table 2:** AI-based interventions and reported improvements in ASD children (2020–2025).

Study (Year)	Country/Sample	Type of Robot/AI Tool	Targeted Skills	Main Outcomes	Measurement Methods
So et al. (2020) [26]	Hong Kong/30 children	Humanoid robot (HUMANE)	Joint attention	↑ Response to joint attention (RJA) and initiation of joint attention (IJA)	Video recordings
Holeva et al. (2022) [28]	Greece/51 children	NAO humanoid robot	Emotion recognition, social interaction	↑ Psychosocial skills, better emotion recognition vs. human-only group	Therapists’ recordings
Ghiglino et al. (2023) [24]	Italy/45 children	iCub (humanoid) vs. Cozmo (toy robot)	Theory of mind, social cognition	iCub: ↑ Theory of mind and prosocial behaviors; Cozmo: no significant change	Standardized tests (NEPSY-II)
Gaitán-Padilla et al. (2022) [27]	Colombia/20 children	CASTOR humanoid robot	Social participation, imitation	↑ maintained social skills	Therapists’ evaluations
Silva et al. (2024) [29]	Portugal and Italy/5 children	NAO + Gaze-tracking + EEG sensors	Attention, engagement	↑ Overall attention but gradual decline in focus on robot	EEG data, gaze-tracking
Chung, Sin and Chow (2024) [20]	Hong Kong/60 children	NAO humanoid robot	Social communication	↑ Social reciprocity and ↓ non-functional behaviors	Parent questionnaires
Van Der Steen et al. (2025) [23]	Netherlands/65 children (ASD + DS)	Robotic dog (WowWee CHiP)	Emotional regulation, confidence	↑ Emotional attunement, live dog > robot dog for emotional regulation	Parent reports
Giannopulu et al. (2018) [25]	France and Japan/40 children	Plant robot “Pekkopa”	Emotional empathy, synchrony	↑ Emotional synchrony with robot vs. human partner	HR and HRV sensors

## Data Availability

No new data were created or analyzed in this study. Data sharing is not applicable to this article.

## Supplementary Materials

The following supporting information can be downloaded at: https://www.mdpi.com/article/10.3390/brainsci16010056/s1, Table S1: PRISMA 2020 Checklist.
